# Rapid model building of β-sheets in electron-density maps

**DOI:** 10.1107/S0907444910000302

**Published:** 2010-02-12

**Authors:** Thomas C. Terwilliger

**Affiliations:** a Los Alamos National Laboratory, Los Alamos, NM 87545, USA

**Keywords:** structure solution, model building, Protein Data Bank, β-strands, *PHENIX*, experimental electron-density maps

## Abstract

A method for rapidly building β-sheets into electron-density maps is presented. β-Strands are identified as tubes of high density adjacent to and nearly parallel to other tubes of density. The alignment and direction of each strand are identified from the pattern of high density corresponding to carbonyl and C^β^ atoms along the strand averaged over all repeats present in the strand. The β-strands obtained are then assembled into a single atomic model of the β-sheet regions. The method was tested on a set of 42 experimental electron-density maps at resolutions ranging from 1.5 to 3.8 Å. The β-­sheet regions were nearly completely built in all but two cases, the exceptions being one structure at 2.5 Å resolution in which a third of the residues in β-sheets were built and a structure at 3.8 Å in which under 10% were built. The overall average r.m.s.d. of main-chain atoms in the residues built using this method compared with refined models of the structures was 1.5 Å.

## Abstract 2

A method for rapid model building of β-sheets at moderate resolution is presented.

## 1. Introduction   

Many methods for the automatic interpretation of macromolecular electron-density maps and model building have recently been developed. These methods address the critical problem of building an atomic model that is consistent with the known sequence of the macromolecule and the expected geometrical features of the polymer. Automated map-interpretation methods are a natural extension of the powerful tools for interactive model building of models into maps [*e.g. O* (Jones *et al.*, 1991 ▶), *MAIN* (Turk, 1992 ▶), *XtalView* (McRee, 1999 ▶) and *Coot* (Emsley & Cowtan, 2004 ▶)], which include semi-automated procedures for the generation of models after the user specifies some information about the chain location or geometry (Oldfield, 1994 ▶; Jones & Kjeldgaard, 1997 ▶; McRee, 1999 ▶). Recently developed highly automated methods for model building of proteins and nucleic acids include procedures that first identify C^α^-atom positions and then extend these to create a model (Oldfield, 2002 ▶, 2003 ▶; Ioerger & Sacchettini, 2003 ▶; Cowtan, 2006 ▶) as well as methods that first find regular secondary structure followed by extension to build loops and other structures (Levitt, 2001 ▶; Terwilliger, 2003 ▶). Other methods begin with the identification of atomic positions and their interpretation in terms of a polypeptide chain (Perrakis *et al.*, 1999 ▶) or begin with some information about the location of the chain and extensive conformational sampling to identify the conformation of the polymer (DePristo *et al.*, 2005 ▶). Recently, probabilistic methods based on the recognition of density patterns in electron-density maps have been developed that extend automated model building to lower resolution ranges than were previously accessible (DiMaio *et al.*, 2007 ▶; Baker *et al.*, 2007 ▶) and methods for building nucleic acids into electron-density maps have been demonstrated (Pavelcik & Schneider, 2008 ▶).

There are several uses for automated model building. The most important of these is to build an atomic model that will form a basis for understanding the biology of the molecule that has been crystallized. An important additional use of automated methods for map interpretation is the evaluation of the quality of electron-density maps during the structure-determination process itself. Although many techniques exist for choosing a high-quality electron-density map (see Terwilliger *et al.*, 2009 ▶), by far the strongest indication that a structure has been ‘solved’ and an accurate electron-density map has been obtained is the ability to interpret the map in terms of an atomic model.

In the context of model building as a tool for map evaluation, the speed of model building is important. The faster the process, the more cases it can be applied to in a short period of time. In particular, the faster the process, the more possibilities for values of parameters in all steps of structure determination can be tested. In our previous template-based methods for main-chain building of protein structures, a key slow step consists of finding which three-amino-acid fragment from a large library best fits the electron density when placed at the tip of a growing chain (Terwilliger, 2003 ▶). Although this step can be optimized, for example by grouping of similar fragments and testing only a small subset of the library, it is intrinsically quite slow.

A much faster overall approach to model building is to look specifically for regular secondary structure in an electron-density map (Jones & Kjeldgaard, 1997 ▶; Cowtan, 1998 ▶, 2008 ▶; Terwilliger, 2003 ▶). As typically more than half the polypeptide chains in protein molecules have either α-­helical or β-­strand structure, a large part of a protein molecule can potentially be built in this way. Furthermore, as both α-helices and β-strands are in many cases quite regular, it is possible to carry out this analysis without considering a large number of different backbone configurations.

One way to look for a specific feature in a map is to use an FFT-based convolution search (Cowtan, 1998 ▶, 2008 ▶). We have previously used such an approach to find α-helices and β-­strands in electron-density maps as the starting point for full model building (Terwilliger, 2003 ▶); however, this process is not as fast as it might be because it requires a separate FFT for each orientation of the search model (*e.g.* a β-strand or α-­helical fragment).

A faster approach might be to identify features that are observable at low resolution or for which the locations can be identified or at least limited and only rotational components need to be sampled (Jones, 2004 ▶; Cowtan, 2008 ▶). For example, it might be possible to place α-helices or β-strands directly in the map and follow this by adjustments of their orientations and positions based on any additional information from the map that has not already been used. We have used this type of approach to build α-­helices into electron-density maps at low resolution (7 Å), where they appear simply as cylinders of density (Terwilliger, 2010 ▶). Higher resolution map information was then used to identify the positioning and direction of the helices. As the initial placement was per­formed with information that was both low resolution and symmetrical (as the α-helices appear as cylinders), it could be carried out rapidly. Although subsequent steps were more time-intensive, they were only applied to the relatively small number of helix placements found, so the entire process was rapid.

Here, we develop a hierarchical method for β-sheet model building in which adjacent strands in a sheet are identified as nearly parallel tubes of density and the direction and register of the strands are identified using density correlations based on the periodicity of β-strands.

## 2. Modeling β-sheets in an electron-density map   

Our approach for modeling the β-sheets in an electron-density map of a protein focuses on speed by examining the map for characteristic features of these structures. The method consists of three steps.

(i) Identification of the location of sheets based on the presence of nearly parallel tubes of density.
(ii) Identification of β-strand alignment and direction using the pattern of high density corresponding to carbonyl and C^β^ atoms along the strand averaged over all repeats present in the strand.
(iii) Assembly of β-strands into a single model.

The result of this process is a model of the β-sheet portions of the structure. It can be used as a starting point for further model building and map interpretation in combination with a model of the α-helical portions of the structure. The steps carried out are described in detail below.

### 2.1. Identification of sheet locations as nearly parallel tubes of density   

**Figure 1:**
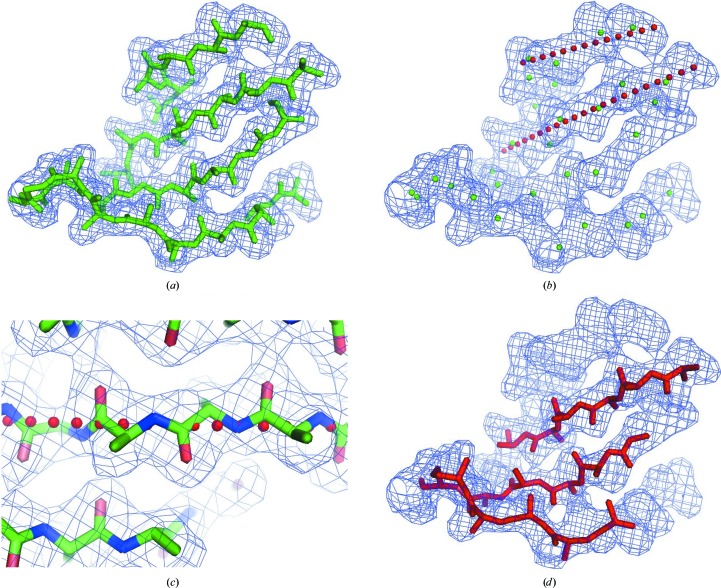
Model β-sheet density and interpretation. (*a*) β-Sheet (from PDB entry 1gba; Mace & Agard, 1995 ▶; with side chains truncated at C^β^ atoms) with model electron density calculated at a resolution of 2.5 Å. (*b*) Electron density with the locations of points along ridgelines of high density marked by green spheres and with red spheres marking the axis of two adjacent curved tubes of density. (*c*) View of model β-sheet showing a β-­strand with carbonyl O atoms pointing up and down towards adjacent strands. (*d*) β-Sheet built using the methods presented here, after the assembly step combining strands obtained by analysis of the map at resolutions of 2.5, 3 and 4 Å. These figures were produced using *PyMOL* (DeLano, 2002 ▶).

Fig. 1 ▶(*a*) illustrates a model of an antiparallel β-sheet and corresponding density at a resolution of 2.5 Å. The first step in our procedure for building β-sheets is the identification of where strands are located in the electron-density map. At moderate resolution (2.5–4 Å) the polypeptide backbone resembles a tube of density and for β-strands the tubes have only a small amount of curvature. In β-sheets these strands are arranged in a nearly parallel or antiparallel fashion, with a small (typically up to about 30°) inclination between adjacent strands. To simplify the identification of strands in a map and to make it as rapid as possible, a pair of strands in a β-sheet is therefore initially considered to consist of two tubes of density that are nearly parallel and that are separated by approximately 4.5 Å at their closest approach. In this analysis, tubes of density are identified, then pairs of nearly parallel tubes are found and finally the tubes are extended into density, allowing curvature of the tubes.

Tubes of density are found in the electron-density map by finding points along ridgelines of high density. Firstly, a set of points in the map that are on ridgelines of high density and are separated typically by 2 Å are identified (green spheres in Fig. 1 ▶ *b*). All pairs of points that are connected by high density are then identified. The criteria for two points being connected are that (i) the density ρ sampled along the line connecting the points has a value of at least ρ_max_ × cut_1_, where ρ_max_ is the higher of the densities at the two end points and cut_1_ typically has a value of 0.5, and (ii) that the mean density ρ_mean_ along the line is at least ρ_max_ × cut_2_, where the typical value of cut_2_ is 0.75. These pairs of connected points and the lines connecting them represent the locations and directions of tubes of density that might be β-strands.

Next, pairs of nearly parallel tubes of density separated by about 4.5 Å are identified. This is performed by finding two nearby nearly parallel pairs of connected points (representing two tubes of density) with no high-density connections between the pairs (such that the density ρ sampled along the line connecting the points has a value of at most ρ_max_ × cut_1_ as defined above). The cosine of the angle between the two tubes of density is typically required to be at least 0.5. The distance between the tubes of density is typically required to be 4.5 ± 2.0 Å at their closest approach. These tubes representing high density in the map are then extended into the available density, allowing the curvature of the tubes to match the high density in the map, as illustrated for the two tubes of density identified by red spheres in Fig. 1 ▶(*b*). To simplify the analysis, this curvature is only allowed in the direction perpendicular to a line connecting the midlines of the two tubes of density at their closest approach. This direction was chosen because the strands in β-sheets typically have a curvature that is roughly perpendicular to the plane of the β-sheets.

This procedure as a whole identifies tubes of density in the electron-density map that have the characteristics expected of a strand that is part of a β-sheet. Additionally, for each tube of density the direction to a neighbouring tube of density is also identified, yielding the expected direction of the carbonyl O atoms relative to the backbone of the β-strand.

To ensure that the tubes of density being considered have a shape that is approximately that expected for a β-strand, each tube of density is scored in two ways. Firstly, the correlation coefficient between the density in the map and an ideal tube of density with a value of 1 along its axis and 0 at a radius of 1.5 Å is estimated. If this is less than the value of cc_strand_min (typically set at 0.5) then the tube of density is discarded as a candidate β-strand position. Otherwise, the score for the tube consists of the mean density along the axis of the tube multiplied by the square root of the length of the tube of density. This is similar to the scoring procedure that we have used previously to evaluate the quality of fit of a model to density (Terwilliger, 2003 ▶).

### 2.2. Identification of β-strand alignment and direction   

Once tubes of density that could represent β-strands have been identified as described above, they are each considered individually for their fit to a model of a β-strand, allowing the curvature of the strand to match the curvature of the tube of density. Fig. 1 ▶(*c*) shows a close-up view of a model strand and of the curved axis of the tube of density corresponding to it. In this step the goal is to start with the density map and the points marking the tube of density and to end with a strand fitted into the density. One way to do this would be to model a strand in all possible positions near the axis of the tube of density and find the one that fits the best. We chose instead to use a faster but less comprehensive method in which the density near the curve marking the tube of density is examined for periodic patterns corresponding to the pattern of carbonyl O and C^β^ atoms along a β-sheet.

Fig. 1 ▶(*c*) illustrates the features of the electron density that we used in this process. The carbonyl O atoms of the strand in the middle of the figure point alternately up towards the β-­strand above it and down towards the β-strand below it in the figure. The C^β^ atoms point alternately into the page and out from it. Note that the C^β^ atoms have a specific relationship to the direction of the chain and the positions of the carbonyl atoms: they are located about two-thirds of the distance from one carbonyl to the next going from right to left (N-terminus to C-terminus) along the chain in the middle of Fig. 1 ▶(*c*). This relationship is what we use to identify the positioning and direction of the β-strand.

The representation of the density for this strand as a tube is marked by the red spheres in Fig. 1 ▶(*c*). Note that the red spheres very nearly coincide with the main-chain atoms of the strand. As the periodicity of the β-strand is known (about 6.7 Å), it is simple and rapid to average the density near the strand over all corresponding locations along the strand. This produces average density for one repeat of the strand. Then, as the direction towards the neighboring strand is already known, the positioning of the carbonyl atoms can readily be identified as being where the density approximately 1.5 Å from the axis of the strand in the direction of the neighbouring strand is maximal (as illustrated by the two carbonyl O atoms pointing up from the middle strand in Fig. 1 ▶ *c*). With the same alignment, another carbonyl O atom points down towards the strand on the other side and this density should be offset by half of the period of the β-strand. In our approach, if these two estimates of the locations of the carbonyls agree to within approximately 1/12 of the period then the identification of the location is considered to be a possible match.

The same approach can then be applied to examine the density, again about 1.5 Å from the axis of the strand, this time in the directions perpendicular to the plane of the β-sheet. This density corresponds to that of the C^β^ atoms and is offset by about 1/3 of a period from that of the carbonyl O atoms (Fig. 1 ▶ *c*). The pattern of high density and direction along the β-strand going from the N-terminus to the C-terminus can now be readily seen. For the β-strand in the middle of Fig. 1 ▶(*c*), starting at the carbonyl O atom pointing up at the right of the figure and moving to the left one atom at a time, it may be seen that the pattern of high density will be (i) up at position 1 (at the carbonyl C atom), (ii) right at atom 3 (the C^β^ atom into the plane of the figure), (iii) down at atom 4 (the next carbonyl), (iv) left at atom 6 (the next C^β^ atom) and then up again at atom 7 (the next carbonyl O atom pointing up). Note that if the strand were in the opposite direction then the pattern would be different. Consequently, the position and direction of the strand can be identified. In our procedure, if all the positions of highest density in this pattern are aligned within a tolerance of 1/12 of the period of the β-strand then the position and direction are considered to be likely to be correct (this would happen in about 1% of cases by chance, as there are two possible directions of the strand, the position of the first carbonyl is defined as the start and the highest density would be within 1/12 of the target position 1/6 of the time for each of the other three atoms).

Given the direction and alignment of a strand as in Fig. 1 ▶(*c*) and the curve corresponding approximately to the main-chain atoms (the red spheres in Fig. 1 ▶ *c*), an idealized β-strand can be placed. In cases where the direction cannot be identified using the method described above, two candidate β-strands are created, one in each direction. Note that if the curvature is substantial then there can be some distortion of the strand.

### 2.3. Assembly of β-strands, elimination of overlaps and joining of adjacent segments   

The analysis described above yields a group of modelled β-­strands that match the electron density. However, these strands may contain overlapping fragments. We use the main-chain assembly routines in the *RESOLVE* software to assemble these fragments and resolve any overlaps (Terwilliger, 2003 ▶). All the β-strands are ranked based on their match to the density using the scoring function described above. β-­Strands that have two or more sequential C^α^ atoms that overlap within about 1 Å are connected into longer chains. The highest scoring chain is selected and all overlapping fragments are deleted. The process is continued until no further fragments with a length of at least four residues are found. Fig. 1 ▶(*d*) shows the result of carrying this out when the strands found from analyses of this map using data to a resolution of 2.5, 3 and 4 Å are merged in this assembly process (the default procedure).

## 3. Application to experimental electron-density maps   

We tested our approach for modeling β-strands using a set of 42 density-modified electron-density maps from the *PHENIX* structure library previously solved by MAD, SAD, MIR and a combination of SAD and SIR procedures with data extending to high resolutions ranging from 1.5 to 3.8 Å. Maps were calculated with the *PHENIX AutoSol* wizard (Adams *et al.*, 2002 ▶; Terwilliger *et al.*, 2009 ▶) using the data that had previously led to refined models for each of the structures considered. Each map was examined for β-strands using the procedure described above.

Table 1 ▶ summarizes the results of these tests. For each structure it shows the number of residues of β-strand in the refined structure (as calculated with *DSSP*; Kabsch & Sander, 1983 ▶), the number of residues of β-strand found, the number of residues found that were correctly identified as β-strand (those for which the C^α^ atom was within 3 Å of a C^α^ atom of a β-strand residue in the refined structure of the protein), the quality of the map (the correlation of the map with a map calculated from the refined model of the structure), the r.m.s. coordinate difference between main-chain atoms in the modeled β-strands compared with those in the refined structure and the correlation between the map and a map calculated from the β-strand model.

On average, 58% of the residues in β-strands as identified by *DSSP* were built using our approach. Of these, 60% of the residues built were in fact in β-strands (the C^α^ atom was within 3 Å of a C^α^ atom of a β-strand residue in the refined structure of the protein). The 40% of the residues built by our procedure that did not match β-strands as identified by *DSSP* were either incorrectly built (*e.g.* traced into helices) or were built into less regular secondary structure (such as loop regions). Therefore, the method built β-sheets reasonably well, but some β-strands were missed and some residues were identified as β-strand that were in fact another type of structure. Overall, the r.m.s.d. between modelled β-strands and refined coordinates was about 1.5 Å. The CPU time required (using 2.9 GHz Intel Xeon processors) to analyze all 42 maps was 66 min or about 0.8 s per residue of β-strand placed.

To compare these results with a standard procedure for automated model building, the same 42 maps were analyzed with the *PHENIX AutoBuild* wizard (Terwilliger *et al.*, 2008 ▶) using one cycle of model building and refinement. The *AutoBuild* wizard built 65% of the residues in β-strands as identified with *DSSP*, with an overall r.m.s.d. (including all main-chain and C^β^ residues built, whether strand or not) of 0.95 Å and required 43 h for the entire set of structures.

One structure for which most β-strand residues were missed was the GroEL structure (PDB entry 1oel; Braig *et al.*, 1995 ▶; Berman *et al.*, 2000 ▶; Bernstein *et al.*, 1977 ▶). This structure has 644 residues in β-strands; however, only 18 of these were found. This structure was at a much lower resolution (3.8 Å) than all the others in this test and the map was of lower quality than most (correlation with a model map of 0.55), suggesting that the method may not work well at lower resolutions or with maps of poor quality.

**Figure 2:**
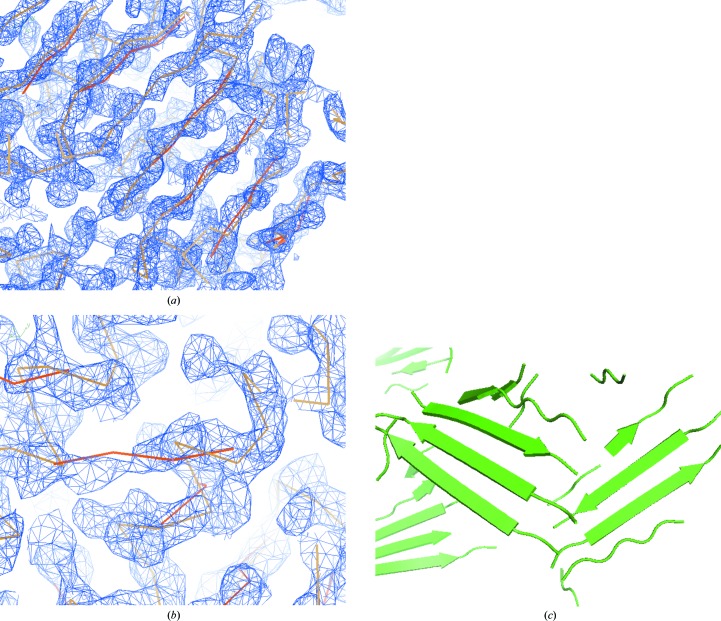
Model building of β-sheets in density-modified experimental electron-density maps. (*a*, *b*) Sections of the electron-density map and model from S-­hydrolase (Turner *et al.*, 1998 ▶) in which the β-sheet model is largely correct (*a*) and incorrect (*b*). The refined model C^α^ trace is shown in light brown and the β-sheet C^α^ trace is shown in dark brown. (*c*) Ribbon diagram of part of the model obtained from structure 1038B at a resolution of 3 Å showing β-­sheets (PDB entry 1lql; Choi *et al.*, 2003 ▶). (*a*) and (*b*) were created with *Coot* (Emsley & Cowtan, 2004 ▶) and (*c*) was created with *PyMOL* (DeLano, 2002 ▶).

In a few cases significantly more β-strand residues were built than were identified by *DSSP*. For example, S-hydrolase (PDB entry 1a7a; Turner *et al.*, 1998 ▶) had 247 β-strand residues built at a resolution of 2.8 Å, but only 83 of these matched a β-­strand residue identified by *DSSP*. Examination of this model showed that much of it was built quite accurately (Fig. 2 ▶ *a*); however, there were other places where β-strands have been built into density that corresponds to α-helices or to side-chain density (Fig. 2 ▶ *b*).

The procedure produced very complete structures of the β-­sheets in many cases. The largest number of β-sheet residues built was for the structure 1038B at a resolution of 3 Å (PDB entry 1lql; Choi *et al.*, 2003 ▶), for which 472 residues of β-sheet were built (and 399 of these matched β-sheet residues identified by *DSSP*) with an r.m.s.d. from the refined model of 1.3 Å. The structure has tenfold NCS but this was not used in the model-building process. A ribbon diagram of this model is shown in Fig. 2 ▶(*c*).

**Figure 3:**
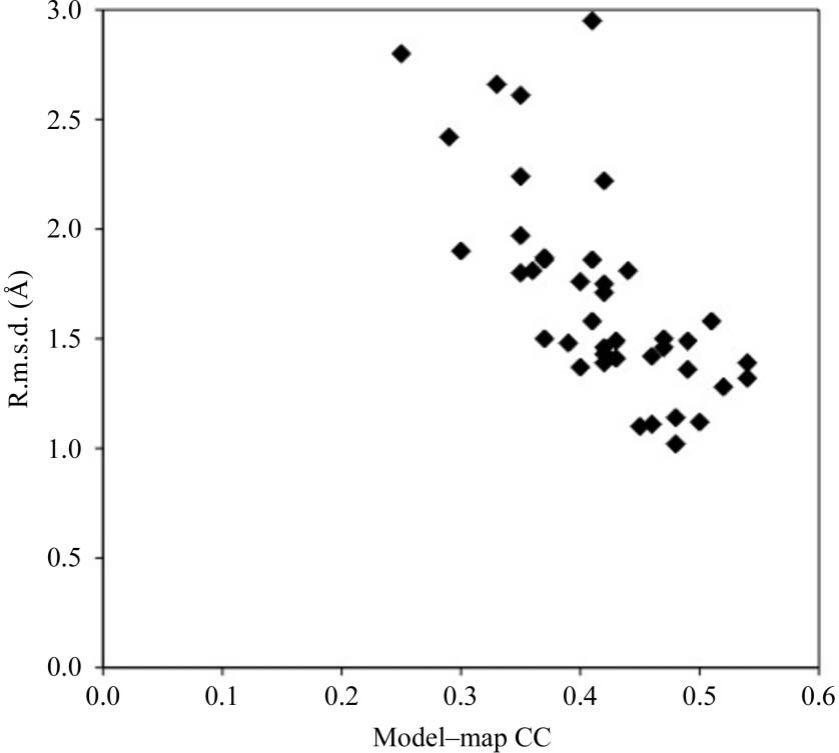
Accuracy of models as a function of map correlation of the models.

It would be useful to have a way to estimate the quality of a model produced with this method in real cases where the structure is not known. One approach to this is simply to calculate the correlation coefficient (CC) between the electron-density map and the β-sheet model, only including points in the map that are near (within 2 Å) of an atom in the model. Fig. 3 ▶ shows that this map correlation does give an indication of the quality of the model (as measured by the r.m.s.d. between model atoms and corresponding atoms in the refined model of the protein).

**Figure 4:**
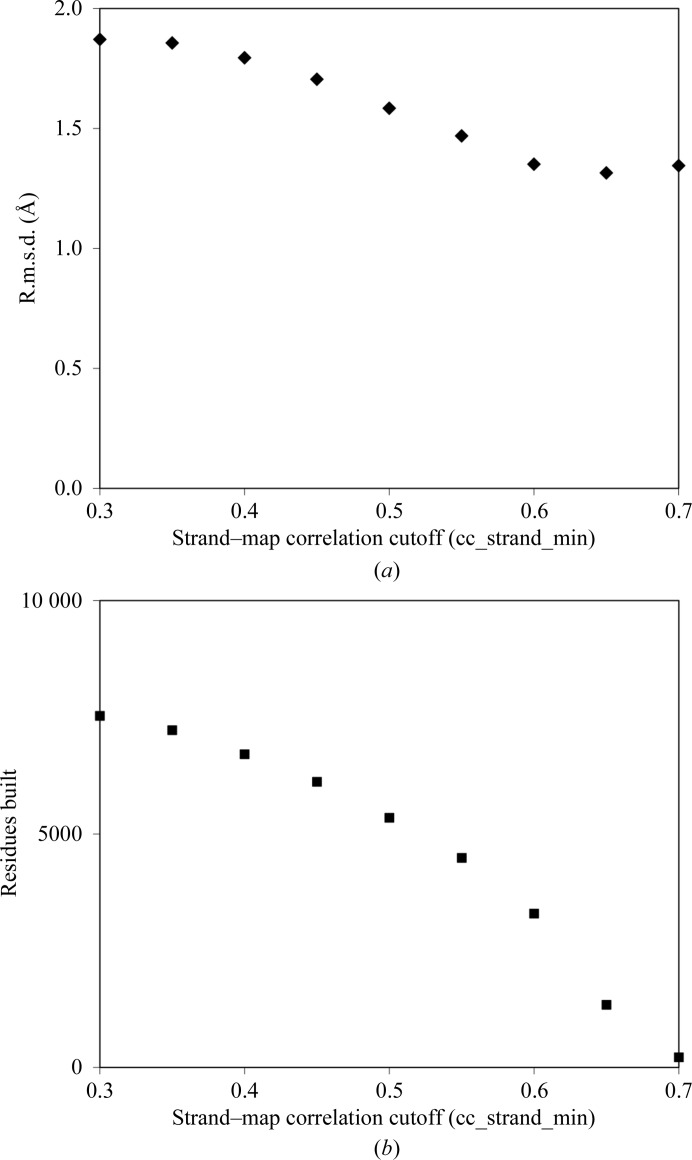
Accuracy of models and residues built as a function of the threshold for strand–map correlation (cc_strand_min). (*a*) The mean r.m.s.d. between β-sheet models and refined structures is shown for the 42 maps in Table 1 ▶. (*b*) The total number of residues built is shown.

One adjustable parameter in this procedure that would be expected to affect both the accuracy of the models and the number of residues built is cc_strand_min, the minimum correlation between the density near a potential strand and that of an idealized tube of density. Fig. 4 ▶(*a*) illustrates the mean value of the r.m.s.d. between main-chain atoms of the β-­sheet models built and the corresponding atoms in refined models as a function of this parameter and Fig. 4 ▶(*b*) illustrates the number of residues built. The accuracy generally improves with increasing stringency, but as expected the number of residues built decreases. Values of cc_strand_min in the range 0.3–0.5 would appear to be reasonable compromises between these competing effects.

## 4. Conclusions   

The procedure that we have developed for modelling β-sheets is quite rapid and reasonably accurate. It identifies most of the β-­sheets in the tests we have carried out. The method does show some overprediction and can accidentally build β-­structure into helical or side-chain density in some cases (Fig. 2 ▶ *b*), but in general it builds β-sheets very well (Fig. 2 ▶ *c*).

Several improvements can readily be imagined for this procedure. One would be to take account of the hydrogen-bonding pattern in β-sheets, which would be expected to improve the register and alignment of the models. Another would be to explicitly look for deviations from regular β-­structure, such as β-bulges or the start of helices, so as to more precisely define the start and end of regular β-strands.

The method may be useful in several ways. Firstly, it can be a good indicator of whether a structure has been solved, as a picture such as that in Fig. 2 ▶(*c*) is not likely to be found unless this is the case. Secondly, the procedure can be part of a rapid scoring procedure for evaluation of electron-density maps by analysis of the regular secondary structure evident in the maps. Lastly, the procedure could be used as part of a more complete model-building process in which the secondary structure built with this method is used as a starting point for chain extension and further model building.

## Figures and Tables

**Table 1:** -Strand identification in experimental electron-density maps

	Residues							
Structure	Total	Strand	Built	Correct	*d*_min_ ()	Map quality (CC to model map)	R.m.s.d. ()	Strandmap CC
RNase P (1nz0; Kazantsev *et al.*, 2003 ▶)	416	88	18	11	1.5	0.53	1.90	0.30
1063B (1lfp; Shin *et al.*, 2002 ▶)	243	57	42	33	1.7	0.68	1.58	0.41
Epsin (1edu; Hyman *et al.*, 2000 ▶)	149	0	15	0	1.8	0.89	2.24	0.35
Isocitrate lyase (1f61; Sharma *et al.*, 2000 ▶)	836	98	161	67	1.8	0.65	1.75	0.42
MBP (1ytt; Burling *et al.*, 1996 ▶)	227	61	60	33	1.8	0.89	1.48	0.39
P9 (1bkb; Peat *et al.*, 1998 ▶)	136	73	59	50	1.8	0.81	1.02	0.48
Penicillopepsin (3app; James Sielecki, 1983 ▶)	323	147	122	94	1.8	0.84	1.10	0.45
Myoglobin (Ana Gonzlez, personal communication)	154	0	17	0	1.9	0.73	3.34	0.24
ROP (1f4n; Willis *et al.*, 2000 ▶)	108	0	20	0	1.9	0.84	2.80	0.25
1167B (1s12; Shin *et al.*, 2005 ▶)	370	108	93	69	2.0	0.72	1.50	0.47
CobD (1kus; Cheong *et al.*, 2002 ▶)	355	56	80	32	2.0	0.80	1.97	0.35
NSF-N (1qcs; Yu *et al.*, 1999 ▶)	195	83	60	42	2.0	0.80	1.50	0.37
Synapsin (1auv; Esser *et al.*, 1998 ▶)	585	179	165	122	2.0	0.78	1.14	0.48
Tryparedoxin (1qk8; Alphey *et al.*, 1999 ▶)	143	33	39	21	2.0	0.79	1.87	0.37
PDZ (1kwa; Daniels *et al.*, 1998 ▶)	174	66	36	30	2.1	0.67	1.41	0.43
Fusion complex (1sfc; Sutton *et al.*, 1998 ▶)	867	0	32	0	2.3	0.73	2.95	0.41
GPATase (1ecf; Muchmore *et al.*, 1998 ▶)	992	223	248	179	2.3	0.82	1.28	0.52
Granulocyte (2gmf; Rozwarski *et al.*, 1996 ▶)	241	20	14	0	2.3	0.62	2.61	0.35
VMP (1l8w; Eicken *et al.*, 2002 ▶)	1141	16	89	8	2.3	0.76	1.80	0.35
Armadillo (3bct; Huber *et al.*, 1997 ▶)	457	0	38	0	2.4	0.86	2.66	0.33
Cyanase (1dw9; Walsh *et al.*, 2000 ▶)	1560	290	294	168	2.4	0.82	1.76	0.40
Mev kinase (1kkh; Yang *et al.*, 2002 ▶)	317	77	91	62	2.4	0.83	1.46	0.47
NSF D2 (1nsf; Yu *et al.*, 1998 ▶)	247	37	70	28	2.4	0.84	1.81	0.44
1102B (1l2f; Shin, Nguyen *et al.*, 2003 ▶)	344	96	82	70	2.5	0.78	1.12	0.50
AEP transaminase (1m32; Chen *et al.*, 2002 ▶)	2169	354	423	264	2.5	0.81	1.46	0.42
FLR (1bkj; Tanner *et al.*, 1996 ▶)	460	62	91	44	2.5	0.77	1.81	0.36
P32 (1p32; Jiang *et al.*, 1999 ▶)	529	144	154	115	2.5	0.86	1.58	0.51
PSD-95 (1jxm; Tavares *et al.*, 2001 ▶)	264	68	69	47	2.5	0.76	1.42	0.46
QAPRTase (1qpo; Sharma *et al.*, 1998 ▶)	1704	324	275	166	2.5	0.71	1.43	0.42
RNase S (1rge; Sevcik *et al.*, 1996 ▶)	192	49	45	25	2.5	0.65	2.42	0.29
Gene V (1vqb; Skinner *et al.*, 1994 ▶)	86	40	24	17	2.6	0.74	1.11	0.46
Rab3A (1zbd; Ostermeier Brnger, 1999 ▶)	301	58	57	37	2.6	0.82	1.49	0.49
GerE (1fse; Ducros *et al.*, 2001 ▶)	384	0	16	0	2.7	0.70	2.22	0.42
CP synthase (1l1e; Huang *et al.*, 2002 ▶)	534	86	138	72	2.8	0.75	1.86	0.41
Rh dehalogenase (1bn7; Newman *et al.*, 1999 ▶)	291	53	67	37	2.8	0.78	1.71	0.42
S-hydrolase (1a7a; Turner *et al.*, 1998 ▶)	861	135	247	83	2.8	0.81	1.86	0.37
UT synthase (1e8c; Gordon *et al.*, 2001 ▶)	990	213	248	157	2.8	0.78	1.49	0.43
1029B (1n0e; Chen *et al.*, 2004 ▶)	1130	232	267	139	3.0	0.73	1.36	0.49
1038B (1lql; Choi *et al.*, 2003 ▶)	1432	483	472	399	3.0	0.71	1.32	0.54
1071B (1nf2; Shin, Roberts *et al.*, 2003 ▶)	801	184	232	143	3.0	0.65	1.39	0.54
Synaptotagmin (1dqv; Sutton *et al.*, 1999 ▶)	275	87	49	29	3.2	0.67	1.39	0.42
GroEL (1oel; Braig *et al.*, 1995 ▶)	3668	644	26	18	3.8	0.55	1.37	0.40
